# Grey Blight Disease Detection on Tea Leaves Using Improved Deep Convolutional Neural Network

**DOI:** 10.1155/2023/7876302

**Published:** 2023-01-17

**Authors:** J. Arun Pandian, Sam Nirmala Nisha, K. Kanchanadevi, Abhay K. Pandey, Samira Kabir Rima

**Affiliations:** ^1^School of Information Technology and Engineering, Vellore Institute of Technology, Vellore, India; ^2^Department of Bio-Technology, Vel Tech Rangarajan Dr.Sagunthala R&D Institute of Science and Technology, Chennai, India; ^3^Department of Computer Science & Engineering, Vel Tech Rangarajan Dr.Sagunthala R&D Institute of Science and Technology, Chennai, India; ^4^Department of Mycology & Microbiology, Tea Research Association, North Bengal Regional R & D Center, Nagrakata-735225, Jalpaiguri, West Bengal, India; ^5^Department of Computer Science and Engineering, American International University, Dhaka, Bangladesh

## Abstract

We proposed a novel deep convolutional neural network (DCNN) using inverted residuals and linear bottleneck layers for diagnosing grey blight disease on tea leaves. The proposed DCNN consists of three bottleneck blocks, two pairs of convolutional (Conv) layers, and three dense layers. The bottleneck blocks contain depthwise, standard, and linear convolution layers. A single-lens reflex digital image camera was used to collect 1320 images of tea leaves from the North Bengal region of India for preparing the tea grey blight disease dataset. The nongrey blight diseased tea leaf images in the dataset were categorized into two subclasses, such as healthy and other diseased leaves. Image transformation techniques such as principal component analysis (PCA) color, random rotations, random shifts, random flips, resizing, and rescaling were used to generate augmented images of tea leaves. The augmentation techniques enhanced the dataset size from 1320 images to 5280 images. The proposed DCNN model was trained and validated on 5016 images of healthy, grey blight infected, and other diseased tea leaves. The classification performance of the proposed and existing state-of-the-art techniques were tested using 264 tea leaf images. Classification accuracy, precision, recall, *F* measure, and misclassification rates of the proposed DCNN are 98.99%, 98.51%, 98.48%, 98.49%, and 1.01%, respectively, on test data. The test results show that the proposed DCNN model performed superior to the existing techniques for tea grey blight disease detection.

## 1. Introduction

Tea farming is an ever-growing industrial sector with increasing production demand as tea is the second most consumed beverage worldwide, next to the water. India is the second largest producer of tea and produced around 1250 million kg in the year 2020 [1]. Tea foliar diseases are of greater concern as they directly affect the harvest, and fungal diseases in particular have a huge impact on the quality and quantity of the produce. In specific, grey blight caused by *Pestalotiopsis theae* is one of the most highly reported diseases from all major tea-growing countries of the world [2]. Mechanical damage to plants incurred by the use of farming equipment initiates infection and disease development. The fungus attacks the maintenance leaves of the tea plant, which ensure nourishment to the tender foliage, indirectly resulting in huge crop loss [3]. The grey blight disease symptoms appear in the middle part of the leaf as brown concentric spots, which later turn grey with brown margins and spread throughout the whole leaf. Detection and diagnosis of symptoms are crucial to controlling the spread of diseases towards sustainable production. The tea cultivation regions are usually large and include mountainous terrains that are difficult to investigate on a routine basis. Concerning tea plantations, conventional methods of disease detection have become ineffective as they rely on intensive manpower and highly specific instruments [4]. Moreover, incorrect diagnosis of the disease leads to inappropriate use of fungicides adding to the production costs and environmental pollution. The above challenges in diagnosing the grey blight infection on tea leaves using conventional techniques are motivated to develop an automatic diagnosis technique.

Computer vision and machine learning techniques have been employed recently in a variety of crops to accurately diagnose diseases and pest attacks based on characteristic symptoms [5–7]. This approach relies on the extraction of features from the leaf images and their identification and classification using an artificial neural network (ANN) [8]. Deep learning, an advanced machine learning technique, which uses deep convolutional neural networks (DCNN) for crop disease identification, is gaining increased application due to its automatic feature extraction ability, accuracy, and robustness in detection [9–11]. For tea disease detection and classification, a few machine learning-based approaches have been employed with considerable performance [5, 12–15].

The major contributions of this research are as follows:

- The grey blight disease detection model for tea leaves was proposed using an improved deep convolutional neural network
- Novel linear bottleneck layers and inverted residual connections were used to design the improved deep convolutional neural network
- The tea grey blight disease dataset was created using 1320 original leaf images of tea crops, including grey blight diseased and nongrey blight diseased leaves
- The dataset size was extended to 5280 images of tea leaves using standard image augmentation techniques such as principal component analysis (PCA), random rotations, random shifts, random flips, resizing, and rescaling
- Hyperparameters of the proposed deep convolutional neural network were optimized using the Bayes grid search technique.
- The proposed DCNN was trained on 5016 images of healthy, grey blight infected, and other diseased tea leaves
- performance of the proposed DCNN was estimated on the test data of 264 images of tea leaves using standard performance metrics such as accuracy, precision, recall, *F* measure, and misclassification rate
- The performance of the proposed DCNN was superior to the recent plant leaf disease detection models and transfer learning techniques for tea grey blight disease detection

The forthcoming sections of the research article are organized as follows.  Section 2 discusses the existing state-of-the-art techniques for leaf disease detection and highlights the significance of the research.  Section 3 elaborates on the tea grey blight disease detection dataset preparation and proposed DCNN model development. In Section 4, the performance of the proposed DCNN model on tea grey blight disease detection is reviewed and compared with the performance of the advanced plant leaf disease detection models and existing transfer learning techniques. Finally, concluding remarks and future directions of the research are discussed in Section 5.

## 2. Related Works

Grey blight, a fungal disease caused by Pestalotiopsis-like species, is a widespread disease affecting tea crops in many tea-growing countries, including India, resulting in huge losses in tea production. The disease typically affects tea leaves from June to September in India. Initially, small brownish spots on the upper surface of the leaves enlarge slowly [16]. These spots may be of various sizes and shapes with an irregular outline. Later, the spot looks dark brown with a greyish appearance at the center and is surrounded by narrow concentric zonation at the leaf margin. The host range of the grey blight pathogen includes guava, strawberry, oil palm, kiwi fruit, mango, pine, and avocado. The authors in [17] found that this disease is caused by Pestalotiopsis, Neopestalotiopsis, and Pseudopestalotiopsis using multilocus DNA sequence-based identification.

This disease has caused around 17–46% crop loss in India and 10–20% yield loss in Japan [3, 18]. The grey blight disease has reduced the tea quality and production by up to 50% in the major tea-growing regions of China and Taiwan [19, 20]. This disease is extensively spreading in the tea gardens of North Bengal of India and other countries such as Korea, China, Kenya, Japan, and Sri Lanka. Advanced artificial intelligence techniques such as machine learning and deep learning performed a significant role in the disease detection of various plant leaves. The DCNNs are the most successful plant disease detection techniques using leaf images [21, 26].  Table 1 compares the numerous detection approaches using machine learning techniques proposed by different articles.

The extensive literature survey shows the significance of DCNN models in tea leaf disease detection. Also, the literature survey identified the following challenges faced by the existing techniques for tea grey blight disease detection. The first challenge is about the visual symptoms. The brown blight, white blight, and bud blight visual symptoms are similar to the grey-blight disease in tea leaves [13, 27–29] that leads to the disease detection models misclassifying the diseases. Second, there is a minimum number of research studies considered to diagnose the grey blight disease in tea crops [30, 31]. However, grey blight is one of the most common yield-restricting diseases of tea crops in India. Finally, the existing techniques were not achieved significant performance in grey blight disease detection. At maximum, the tea leaf disease detection model achieved 94.45% of classification accuracy [30]. The above studies show the significance of proposing a novel approach to the diagnosis of grey blight disease with better performance than the existing works. Also, the approach should understand the difference between grey blight disease, brown blight, white blight, and bud blight disease symptoms. The development and training process of the proposed grey blight disease detection model is discussed in Section 3.

## 3. Materials and Methods

This section describes the proposed DCNN model and dataset for grey blight disease detection.  Section 3.1 explains the grey blight disease dataset collection and preparation. The construction and training process of the proposed DCNN model is explained in Section 3.2.

### 3.1. Data Collection and Preparation

**Figure 1:**
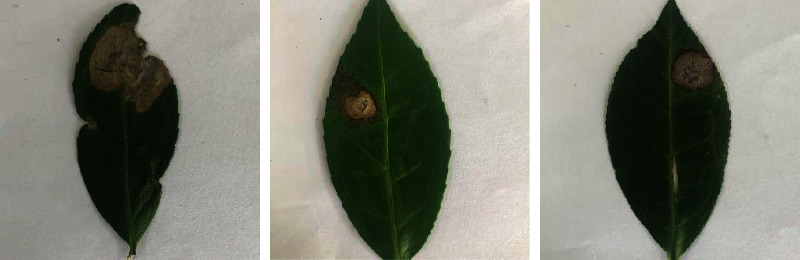
Sample images from the tea grey blight disease dataset.

In the present study, tea gardens located in North Bengal, India, were visited during 2020-2021 to examine the disease pattern of grey blight. Almost all 27 tea gardens visited were infected with grey blight disease with different symptoms. The image of the symptoms was taken using a Canon digital single-lens reflex camera of 500 pixels. There are 1320 images of healthy, grey blight diseased and other diseased leaves captured for preparing the tea grey blight disease dataset.  Figure 1 shows the sample leaf images of the captured data.

**Figure 2:**
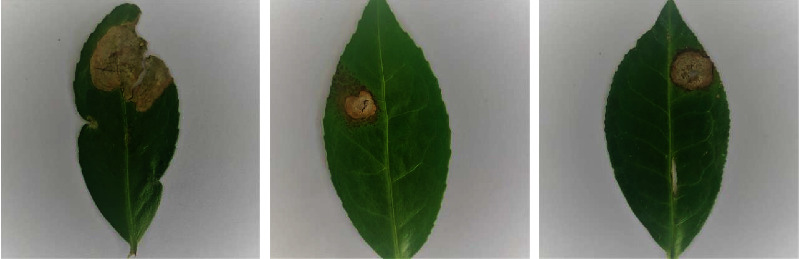
Sample PCA color-augmented images.

The data augmentation techniques, such as the principal component analysis (PCA) color, random rotations, random shifts, random flips, resizing, and rescaling, were used to create 3960 images in the tea grey blight disease dataset. The PCA is an unsupervised machine learning technique that was generally used for clustering data. Recently, PCA techniques have been used as an augmentation technique in various image classification applications [32]. The PCA color-augmented images of the sample images from the tea grey blight disease dataset are shown in Figure 2.

The augmented images were used to increase the number of data and balance the data count in each class of the tea grey blight dataset. The augmented data are added only to the training and validation datasets. The tea grey blight disease dataset was split into training, validating, and testing datasets. The dataset is split up for training, validation, and testing processes as illustrated in Table 2.

The training and validation datasets were used for the training and validation process of the proposed DCNN and standard transfer learning techniques. Each class in the training data consists of 1584 tea leaves images. The test data were used for testing the performance of the proposed DCNN and existing transfer learning techniques. The test dataset contains only originally collected data. The subsequent subsection explains the layered architecture and training process of the proposed DCNN model for the tea grey blight detection task.

### 3.2. Classification Model Design and Training

**Figure 3:**
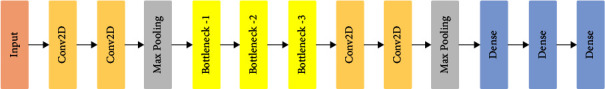
Layered architecture of proposed DCNN.

The proposed DCNN model consists of a sequence of 13 layers. The proposed DCNN model design was inspired by the architectures of MobileNet and VGG19 Net [33, 34]. It uses the inverted residual connections and bottleneck layers from MobileNet and convolutional layer pairs and the downsampling process from VGG19Net.  Figure 3 shows the layered architecture of the proposed DCNN model.

**Figure 4:**
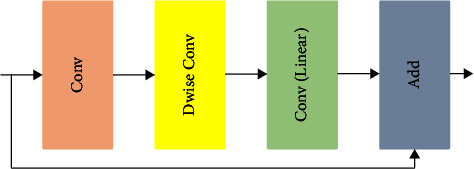
Internal layers of bottleneck block.

The first layer in the proposed DCNN model was named the input layer; it performs resizing the input image dimensions to 224 ∗ 224 ∗ 3 pixels. The resized image was forwarded as an input to the pair of two-dimensional convolutional (Conv2D) layers with a filter size of 128, kernel size of 3 ∗ 3 with a stride value of 1 ∗ 1, and a ReLU activation function. The max-pooling layer was introduced as a fourth layer of the proposed DCNN model to down the sampling size of the convolutional layer output. The max-pooling layer uses a 2 ∗ 2-sized kernel with 1 ∗ 1-sized strides. The downsampled data were forwarded to the sequence of three bottleneck blocks.  Figure 4 illustrates the internal layers of the bottleneck block.

Each bottleneck block consists of four internal layers, such as convolutional (Conv) layers, depthwise convolutional (Dwise Conv) layers, linear convolutional layers, and adder (Add) layers. The Conv layer performs the convolutional operation for extracting the feature information from the output data of the previous layer. The Dwise Conv layers perform the Conv operation on the output of the conv layer with a single filter for all the channels. The Dwise Conv layers require very less computational process compared with the traditional Conv layers. The linear Conv layer was introduced after the Dwise Conv in the bottleneck layer of the proposed DCNN model. The linear Conv layer implements the convolution operations using linear activation functions. The Add layer combines the output data of the linear Conv layer and the input data of the current bottleneck layer in the model. All the Conv layers in the bottleneck block used the kernel size of 3 ∗ 3, the stride value of 1 ∗ 1, and the activation function of ReLU.

**Figure 5:**
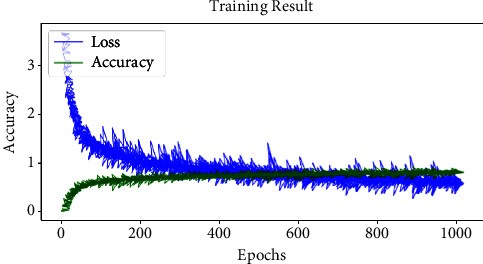
Training performance of proposed DCNN.

In addition, the output data of bottleneck block 3 was forwarded to the pair of Conv layers. The Conv layer pair performs a convolutional operation with a filter size of 64, kernel size of 3 ∗ 3, and stride value of 1 ∗ 1. The max-pooling layer was introduced after the pair of Conv layers in the network to perform downsampling. The max-pooling layer uses a filter size of 2 ∗ 2 with a stride value of 1 ∗ 1. The downsampled data were forwarded to the sequence of three fully connected layers (Dense) with filter sizes of 1024, 512, and 3, respectively. The final dense layer classifies the input data into three classes using the softmax activation function. The Bayes grid search technique was used to optimize the parameter values for the proposed DCNN model. The Bayes grid search technique identified the optimized batch size as 32, the loss function as categorical cross-entropy, the optimizer as Adam, and the learning rate as 0.001 for the proposed DCNN model. The proposed DCNN was trained on the grey blight dataset using optimized hyperparameters up to 1000 epochs. The training progress of the proposed DCNN model is shown in Figure 5.

**Figure 6:**
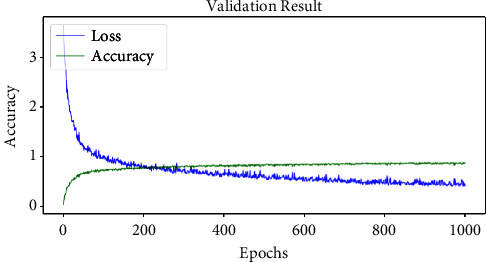
Validation performance of proposed DCNN.

The proposed DCNN model achieved a training accuracy of 99.53% and a training loss of 0.042 on the final training epoch. Also, the model performance was validated using a validation dataset.  Figure 6 illustrates the validation epoch-wise validation performance of the proposed DCNN model on the grey-blight disease dataset.

The proposed DCNN model achieved the validation accuracy and loss on the final training epoch of 99.27% and 0.096, respectively. The trained DCNN model architecture and weights were stored for the testing and deployment processes. Section 4 discusses the test performance of the proposed DCNN model on the test dataset. Also, it compares the performance of the proposed DCNN model with recent transfer learning techniques.

## 4. Results and Discussion

**Figure 7:**
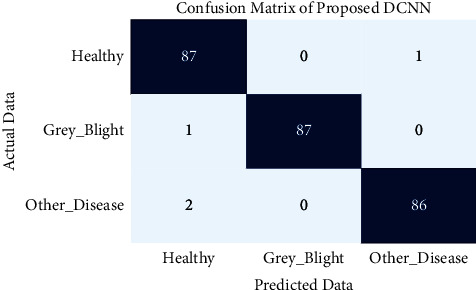
Confusion matrix of proposed DCNN.

This section compares the performance of the proposed DCNN model with recent transfer learning techniques for grey blight disease detection. The recent transfer learning techniques are AlexNet, DenseNet201, InceptionV3Net, MobileNetV2, NASNet Large, ResNet152, VGG19Net, and XceptionNet. There 264 tea leaf images were used for testing the performance of the proposed and existing models.  Figure 7 shows the code-generated confusion matrix of the proposed DCNN on the test dataset.

The test outcome of the proposed DCNN as True Positive (TP), True Negative (TN), False Positive (FP), and False Negative (FN) is shown in Table 3. The code generated a confusion matrix, and scores of the existing transfer learning techniques are included in the Supplementary Materials (available here).

Classification accuracy, precision, recall, *F* measure, misclassification rate, and receiver operating characteristic (ROC) curve were used as metrics for estimating the performance of the grey blight leaf disease detection models in tea leaves. The TP, TN, FP, and FN were used to calculate the performance metric scores. Equations (1–5) show the standard formulas to calculate the accuracy, precision, recall, *F* measure, and misclassification rate [35]:

(1) $$\begin{array}{c} Accuracy=\frac{TP+TN}{TP+TN+FP+FN}\text{,} \end{array}$$

(2) $$\begin{array}{c} Precision=\frac{TP}{TP+FP}\text{,} \end{array}$$

(3) $$\begin{array}{c} Recall=\frac{TP}{TP+FN}\text{,} \end{array}$$

(4) $$\begin{array}{c} F-Measure=\frac{2*Precision*\;Recall}{Precision+Recall}\text{,} \end{array}$$

(5) $$\begin{array}{c} Misclassification\;Rate=\frac{FP+PN}{FP+FN+TP+TN}\text{.} \end{array}$$

Table 4 illustrates the class-wise performance metrics score and weighted average performance score of the proposed DCNN model on test data.

**Figure 8:**
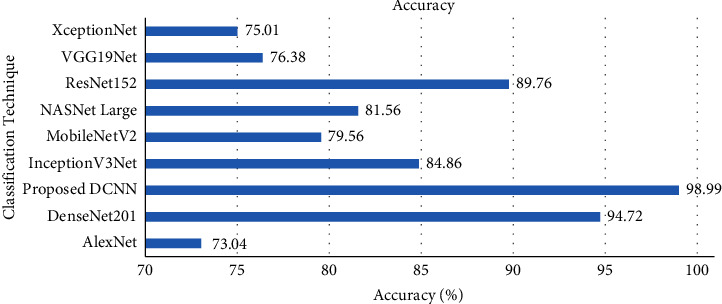
Accuracy comparison with transfer learning techniques.

The average performance metric scores of the existing transfer learning techniques are given in the supplementary materials. The average performance metric scores of the proposed model were compared with the transfer learning techniques. At first, the average classification accuracy of the proposed DCNN model was compared with the transfer learning techniques, and the result is illustrated in Figure 8.

**Figure 9:**
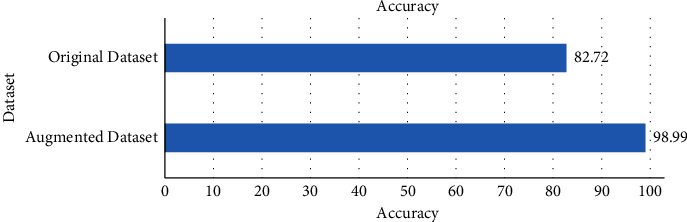
Accuracy comparison of the augmented and original datasets.

The classification accuracy comparison result shows that the proposed DCNN model achieved better accuracy than the AlexNet, DenseNet201, InceptionV3Net, MobileNetV2, NASNet Large, ResNet152, VGG19Net, and XceptionNet models. The proposed DCNN model achieved a classification accuracy of 98.99% in test data, which is 4.27% higher than the second-best performed model named DenseNet201. Also, the proposed DCNN model was trained on the original and augmented datasets separately. The test accuracy of the original dataset trained and augmented dataset trained DCNN models is compared in Figure 9. The comparison result proves that the augmented dataset increased the performance of the proposed DCNN model by 16.27% from the original dataset trained model.

**Figure 10:**
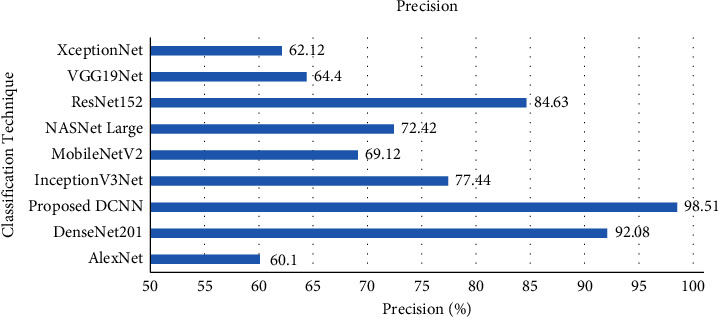
Precision comparison with transfer learning techniques.

As well, the average precision of the proposed DCNN model is compared with the transfer learning techniques in Figure 10. The comparison result shows that the average precision of the proposed DCNN model achieved 98.51% on the grey blight leaf disease dataset. The precision score of the proposed DCNN model was 6.43% higher than the DenseNet201 model and much higher than other transfer learning techniques.

**Figure 11:**
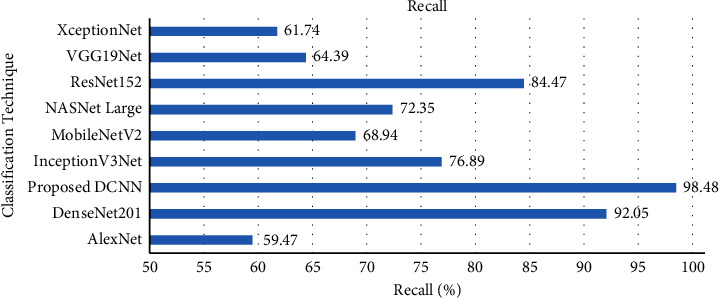
Recall comparison with transfer learning techniques.

In addition, the proposed DCNN model achieved a recall score of 98.48% on the grey blight test dataset. The comparison of the recall score of the proposed DCNN and existing techniques is shown in Figure 11. The comparison result illustrates that the proposed DCNN model achieved a better recall score than the AlexNet, DenseNet201, InceptionV3Net, MobileNetV2, NASNet Large, ResNet152, VGG19Net, and XceptionNet models.

**Figure 12:**
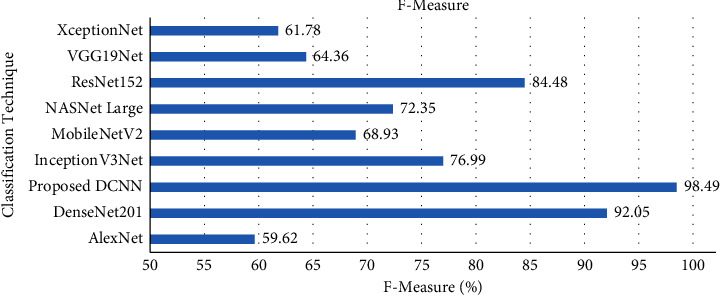
*F* measure comparison with transfer learning techniques.

Furthermore, the *F* measure of the proposed DCNN and existing models on the grey blight dataset is illustrated in Figure 12. The proposed DCNN model achieved an *F* measure score of 98.48% on test data. The *F* measure of the proposed DCNN model was better than the transfer learning techniques such as AlexNet, DenseNet201, InceptionV3Net, MobileNetV2, NASNet Large, ResNet152, VGG19Net, and XceptionNet. The *F* measure score of the proposed DCNN model was 6.44% higher than the Dense201 model on test data.

**Figure 13:**
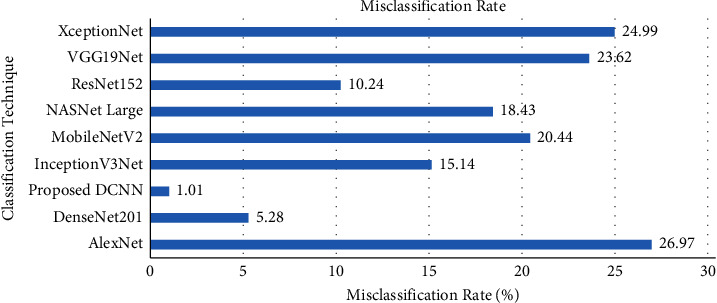
Misclassification rate comparison with transfer learning techniques.

The misclassification rate is also one of the important performance metrics, which predicts the percentage of samples that were incorrectly classified by the models.  Figure 13 shows the misclassification rate of the proposed DCNN and existing models on test data. The proposed DCNN models reached a 1.01% of misclassification rate on the grey blight dataset. The misclassification rate of the proposed DCNN model was very lesser than other techniques.

**Figure 14:**
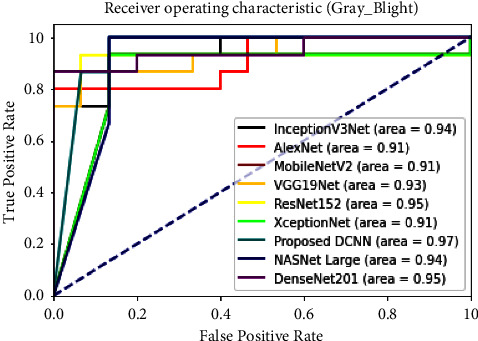
AUC-ROC comparison with transfer learning techniques.

The Receiver operating characteristic (ROC) curve represents the performance of the classification models on all the classification thresholds [36]. The RoC curves of the proposed and transfer learning techniques for individual classes in the dataset are shown in Figure 14. The area under the ROC curve of the proposed DCNN on the grey blight disease class was 97%. The comparison graph shows that the area under the ROC curve of the proposed model for the grey blight disease class was higher than the transfer learning techniques.

The comparison results show that the classification accuracy, precision, recall, *F*- measure, and RoC of the proposed DCNN model was superior to recent transfer learning techniques such as AlexNet, DenseNet201, InceptionV3Net, MobileNetV2, NASNet Large, ResNet152, VGG19Net, and XceptionNet.

**Figure 15:**
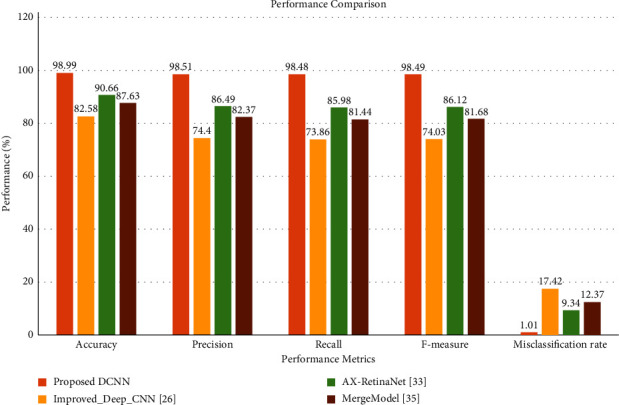
Performance comparison with existing tea leaf disease detection techniques.

Similarly, the classification performance of the proposed DCNN model was compared with the existing state-of-the-art tea leaf disease detection models such as Improved_Deep_CNN [37], AX-RetinaNet [31], and MergeModel [38]. The existing models were trained and tested on the tea grey blight disease dataset.  Figure 15 shows the performance comparison of the proposed and existing models on grey blight disease detection in tea leaves.

The comparison result illustrates that the proposed DCNN model performed better than the existing tea leaf disease detection model on the grey blight disease detection task. The confusion matrix and class-wise performance of the existing techniques are given in the supplementary materials. The subsequent section discussed the conclusions and future directions of the research on grey blight disease detection in tea crops.

## 5. Conclusions

Grey blight is one of the most yield-affecting diseases of tea crops worldwide. Tea plantation regions are generally surface areas and mountainous terrains. It is difficult to diagnose the disease in the entire area manually. This article proposed a novel deep convolutional neural network (DCNN) for the automatic diagnosis of grey blight disease in tea crops. The tea leaf data were collected for the training and testing process of the DCNN model in the North Bengal region of India. Also, the data augmentation techniques were used to increase the number of samples in the training dataset. Principal component analysis (PCA), random rotations, random shifts, random flips, resizing, and rescaling were used to produce the augmented images for the training data. The dataset consists of three classes such as grey blight, healthy, and other diseases. There are 4752 images used for the training and 264 images used for the validation process of the proposed DCNN model, respectively. The model was trained and validated on the dataset till 1000 training epochs. The model achieved an accuracy of 99.27% on the validation data. The performance of the DCNN model was tested using test data from 264 images after the completion of the training process. The test performance of the proposed model was compared with AlexNet, DenseNet201, InceptionV3Net, MobileNetV2, NASNet Large, ResNet152, VGG19Net, and XceptionNet. Also, the proposed DCNN model was compared with the existing state-of-the-art tea leaf disease detection models. The comparison result shows that the classification performance of the proposed DCNN was superior to the transfer learning techniques and existing tea leaf disease detection models on grey blight detection in tea crops. The DCNN model achieved the classification accuracy, precision, recall, and *F* measure which are 98.99%, 98.51%, 98.48%, and 98.49%, respectively. The proposed DCNN achieved a very less misclassification rate of 1.01%, which was less than other techniques. In the future, the proposed model will be deployed in the unmanned aerial vehicle for diagnosing grey blight disease in larger surface areas and mountainous terrains. Diagnosing other yield-controlling diseases of the tea crops and enhancing the classification performance of the proposed model are also the future directions of the research.

## Figures and Tables

**Table 1:** Comparison of existing tea leaf disease detection techniques.

Article	Year	Diseases	Number of		Methodology	Accuracy (%)
			Classes	Images		
24	2018	Brown blight, blister blight, and algal leaf spot	4	1223	Custom DCNN	81.08
25	2019	Anthracnose	2	100	Iterative self-organizing data analysis technique (ISODATA)	98
26	2019	Leaf blight, bud blight, and red scab	4	36	Custom DCNN	92.5
27	2020	Brown blight, blister blight, and leaf spot	4	1822	Faster region-based convolutional neural network (Faster RCNN)	89.4
28	2021	Algal leaf spot, grey blight, white spot, brown blight, red scab, bud blight, and grey blight	8	860	Custom DCNN	94.45
29	2021	Red rust, red spider, thrips, helopeltis, and sunlight scorching	6	1000	Principal component analysis (PCA) and multiclass support vector machine (SVM)	83
30	2021	Leaf blight	2	970	Retinex algorithm and faster RCNN	84.45
31	2021	Brown blight, blister blight, and leaf spot	4	4295	Cascade RCNN (CRCNN)	76.6
32	2022	Leaf spot, rhizome rot, powdery mildew, and leaf blotch	5	630	Hybrid filter and support vector machine	92.84
33	2022	Red leaf spot, algal leaf spot, bird's eyespot, grey blight, white spot, anthracnose, and brown blight	8	885	Improved retina-net	93.83
34	2022	Blister blight	2	60000	Deep hashing with integrated autoencoders (DHIA)	98.5
35	2022	White scab, leaf blight, red scab, and sooty mould	5	634	Custom DCNN and generative adversarial network (GAN)	93.24

**Table 2:** Data split up for training, validation, and testing.

Dataset	Number of images
Training dataset	4752
Validation dataset	264
Test dataset	264
Total images	5280

**Table 3:** Confusion matrix score of DCNN.

Class	True positive	True negative	False positive	False negative
Healthy	87	173	3	1
Grey blight	87	176	0	1
Other disease	86	175	1	2

**Table 4:** Class-wise performance of DCNN.

Class	Accuracy	Precision	Recall	*F* measure	Misclassification rate
Healthy	98.48	96.67	98.86	97.75	1.52
Grey blight	99.62	100	98.86	99.43	0.38
Other diseases	98.86	98.85	97.73	98.29	1.14
Average	98.99	98.51	98.48	98.49	1.01

## Data Availability

The data used to support the findings of this study can be obtained from the corresponding author upon request.

## Supplementary Materials

Supplementary Materials The experimental results of the transfer learning and existing tea leaf disease detection models were illustrated in the supplementary file. Figure 1 shows the confusion matrix of the DenseNet201 model for tea grey blight disease detection. Table 1 illustrates the TP, TN, FP, and FN of the DenseNet201. And, table 2 represents the class-wise and average accuracy, precision, recall, *F* measure, and misclassification rate of the DenseNet201. Figure 2, Table 3, and Table 4 illustrate the confusion matrix, confusion matrix score, and performance of the ResNet152 model, respectively. The confusion matrix of the InceptionV3Net is shown in Figure 3. Table 5 shows the TP, TN, FP, and FN scores of the InceptionV3Net. The performance of the InceptionV3Net on tea grey blight disease detection is illustrated in Table 6. The confusion matrix, confusion matrix score, and class-wise performance of the NASNet Large were shown in Figure 4, Table 7, and Table 8, respectively. Figure 5 depicts the MobileNetV2 confusion matrix. The confusion matrix scores of the MobileNetV2 are shown in Table 9. Table 10 shows how effective the MobileNetV2 is at detecting tea grey blight. Figure 6, Table 11, and Table 12 illustrate the confusion matrix, confusion matrix score, and performance of the VGG19Net model, respectively. The confusion matrix of the XceptionNet for grey blight detection is shown in figure 7. The scores of the confusion matrix were represented in Table 13. The classification performance of XceptionNet is illustrated in Table 14. The confusion matrix, confusion matrix score, and class-wise performance of the AlexNet were shown in Figure 8, Table 15, and Table 16, respectively. Moreover, the confusion matrices of the existing tea leaf disease detection models such as AX-RetinaNet, merge model, and Improved_Deep_CNN were shown in Figures 9, 10, and 11, respectively. Tables 17 and 18 illustrate the confusion matrix score and class-wise performance of the AX-RetinaNet. Table 19 shows the confusion matrix score of the merge model. The class-wise performance of the model was shown in Table 20. Tables 21 and 22 illustrate the confusion matrix scores and class-wise performance of the Improved_Deep_CNN, respectively.
