# Supplementary material for: Grey Blight Disease Detection on Tea Leaves Using Improved Deep Convolutional Neural Network
Source: Comput Intell Neurosci. 2023 Jan 17;2023:7876302. doi: 10.1155/2023/7876302 (PMC9873464; doi:10.1155/2023/7876302)
Supplement: Supplementary Materials — The experimental results of the transfer learning and existing tea leaf disease detection models were illustrated in the supplementary file. Figure 1 shows the confusion matrix of the DenseNet201 model for tea grey blight disease detection. Table 1 illustrates the TP, TN, FP, and FN of the DenseNet201. And, table 2 represents the class-wise and average accuracy, precision, recall, F measure, and misclassification rate of the DenseNet201. Figure 2, Table 3, and Table 4 illustrate the confusion matrix, confusion matrix score, and performance of the ResNet152 model, respectively. The confusion matrix of the InceptionV3Net is shown in Figure 3. Table 5 shows the TP, TN, FP, and FN scores of the InceptionV3Net. The performance of the InceptionV3Net on tea grey blight disease detection is illustrated in Table 6. The confusion matrix, confusion matrix score, and class-wise performance of the NASNet Large were shown in Figure 4, Table 7, and Table 8, respectively. Figure 5 depicts the MobileNetV2 confusion matrix. The confusion matrix scores of the MobileNetV2 are shown in Table 9. Table 10 shows how effective the MobileNetV2 is at detecting tea grey blight. Figure 6, Table 11, and Table 12 illustrate the confusion matrix, confusion matrix score, and performance of the VGG19Net model, respectively. The confusion matrix of the XceptionNet for grey blight detection is shown in figure 7. The scores of the confusion matrix were represented in Table 13. The classification performance of XceptionNet is illustrated in Table 14. The confusion matrix, confusion matrix score, and class-wise performance of the AlexNet were shown in Figure 8, Table 15, and Table 16, respectively. Moreover, the confusion matrices of the existing tea leaf disease detection models such as AX-RetinaNet, merge model, and Improved_Deep_CNN were shown in Figures 9, 10, and 11, respectively. Tables 17 and 18 illustrate the confusion matrix score and class-wise performance of the AX-RetinaNet. Table 19 shows th [file 7876302.f1.docx]

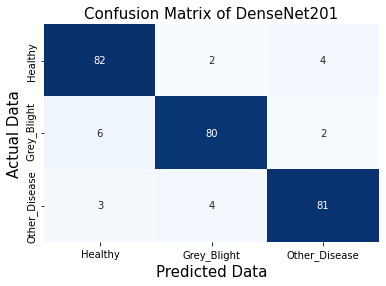


**Fig. 1:** Confusion matrix of DenseNet201

**Table 1:** Confusion matrix score of DenseNet201

|  | **TP** | **TN** | **FP** | **FN** |
| --- | --- | --- | --- | --- |
| **Healthy** | 82 | 167 | 9 | 6 |
| **Grey Blight** | 80 | 170 | 6 | 8 |
| **Other Disease** | 81 | 170 | 6 | 7 |

**Table 2:** Class-wise performance of DenseNet201

|  | **Accuracy** | **Precision** | **Recall** | **F-Measure** | **Misclassification Rate** |
| --- | --- | --- | --- | --- | --- |
| **Healthy** | 94.38 | 90.11 | 93.18 | 91.62 | 5.62 |
| **Grey Blight** | 94.7 | 93.02 | 90.91 | 91.95 | 5.3 |
| **Other Disease** | 95.08 | 93.1 | 92.05 | 92.57 | 4.92 |
| **Average** | **94.72** | **92.08** | **92.05** | **92.05** | **5.28** |


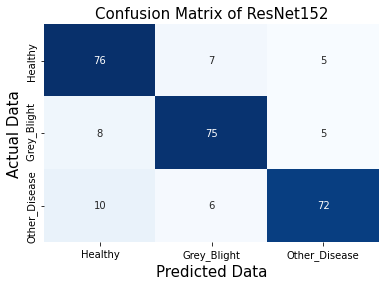


**Fig. 2:** Confusion matrix of ResNet152

**Table 3:** Confusion matrix score of ResNet152

|  | **TP** | **TN** | **FP** | **FN** |
| --- | --- | --- | --- | --- |
| **Healthy** | 76 | 158 | 18 | 12 |
| **Grey Blight** | 75 | 163 | 13 | 13 |
| **Other Disease** | 72 | 166 | 10 | 16 |

**Table 4:** Class-wise performance of ResNet152

|  | **Accuracy** | **Precision** | **Recall** | **F-measure** | **Misclassification Rate** |
| --- | --- | --- | --- | --- | --- |
| **Healthy** | 88.97 | 80.85 | 86.36 | 83.52 | 11.03 |
| **Grey Blight** | 90.15 | 85.23 | 85.23 | 85.23 | 9.85 |
| **Other Disease** | 90.15 | 87.8 | 81.82 | 84.71 | 9.85 |
| **Average** | **89.76** | **84.63** | **84.47** | **84.48** | **10.24** |


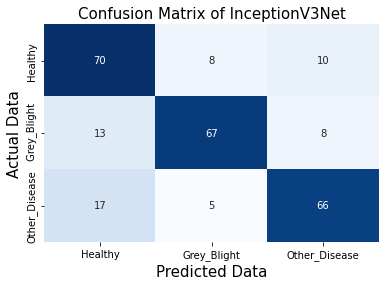


**Fig. 3:** Confusion matrix of InceptionV3Net

**Table 5:** Confusion matrix score of InceptionV3Net

|  | **TP** | **TN** | **FP** | **FN** |
| --- | --- | --- | --- | --- |
| **Healthy** | 70 | 146 | 30 | 18 |
| **Grey Blight** | 67 | 163 | 13 | 21 |
| **Other Disease** | 66 | 158 | 18 | 22 |

**Table 6:** Class-wise performance of InceptionV3Net

|  | **Accuracy** | **Precision** | **Recall** | **F-measure** | **Misclassification Rate** |
| --- | --- | --- | --- | --- | --- |
| **Healthy** | 82.61 | 70 | 79.55 | 74.47 | 17.39 |
| **Grey Blight** | 87.12 | 83.75 | 76.14 | 79.76 | 12.88 |
| **Other Disease** | 84.85 | 78.57 | 75 | 76.74 | 15.15 |
| **Average** | **84.86** | **77.44** | **76.89** | **76.99** | **15.14** |


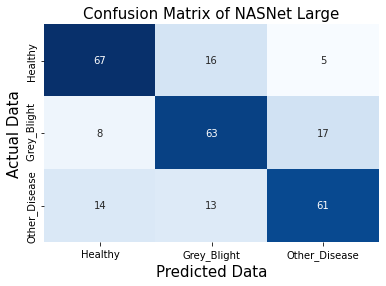


**Fig. 4:** Confusion matrix of NASNet Large

**Table 7:** Confusion matrix score of NASNet Large

|  | **TP** | **TN** | **FP** | **FN** |
| --- | --- | --- | --- | --- |
| **Healthy** | 67 | 154 | 22 | 21 |
| **Grey Blight** | 63 | 147 | 29 | 25 |
| **Other Disease** | 61 | 154 | 22 | 27 |

**Table 8:** Class-wise performance of NASNet Large

|  | **Accuracy** | **Precision** | **Recall** | **F-measure** | **Misclassification Rate** |
| --- | --- | --- | --- | --- | --- |
| **Healthy** | 83.71 | 75.28 | 76.14 | 75.71 | 16.29 |
| **Grey Blight** | 79.55 | 68.48 | 71.59 | 70 | 20.45 |
| **Other Disease** | 81.44 | 73.49 | 69.32 | 71.35 | 18.56 |
| **Average** | **81.57** | **72.42** | **72.35** | **72.35** | **18.43** |


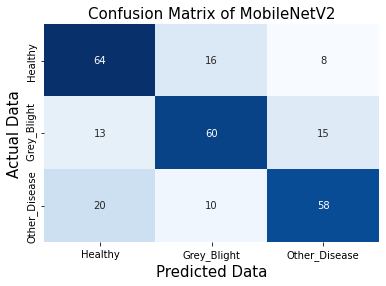


**Fig. 5:** Confusion matrix of MobileNetV2

**Table 9:** Confusion matrix score of MobileNetV2

|  | **TP** | **TN** | **FP** | **FN** |
| --- | --- | --- | --- | --- |
| **Healthy** | 64 | 143 | 33 | 24 |
| **Grey Blight** | 60 | 150 | 26 | 28 |
| **Other Disease** | 58 | 153 | 23 | 30 |

**Table 10:** Class-wise performance of MobileNetV2

|  | **Accuracy** | **Precision** | **Recall** | **F-measure** | **Misclassification Rate** |
| --- | --- | --- | --- | --- | --- |
| **Healthy** | 79.2 | 65.98 | 72.73 | 69.19 | 20.8 |
| **Grey Blight** | 79.55 | 69.77 | 68.18 | 68.97 | 20.45 |
| **Other Disease** | 79.92 | 71.6 | 65.91 | 68.64 | 20.08 |
| **Average** | **79.56** | **69.12** | **68.94** | **68.93** | **20.44** |


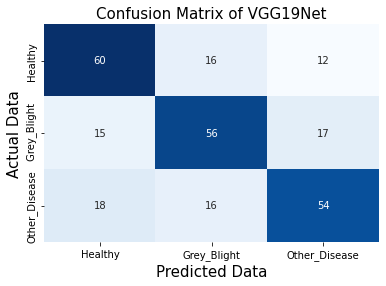


**Fig. 6:** Confusion matrix of VGG19Net

**Table 11:** Confusion matrix score of VGG19Net

|  | **TP** | **TN** | **FP** | **FN** |
| --- | --- | --- | --- | --- |
| **Healthy** | 60 | 143 | 33 | 28 |
| **Grey Blight** | 56 | 144 | 32 | 32 |
| **Other Disease** | 54 | 147 | 29 | 34 |

**Table 12:** Class-wise performance of VGG19Net

|  | **Accuracy** | **Precision** | **Recall** | **F-measure** | **Misclassification Rate** |
| --- | --- | --- | --- | --- | --- |
| **Healthy** | 77.24 | 64.52 | 68.18 | 66.3 | 22.76 |
| **Grey Blight** | 75.76 | 63.64 | 63.64 | 63.64 | 24.24 |
| **Other Disease** | 76.14 | 65.1 | 61.36 | 63.16 | 23.86 |
| **Average** | **76.38** | **64.4** | **64.39** | **64.36** | **23.62** |


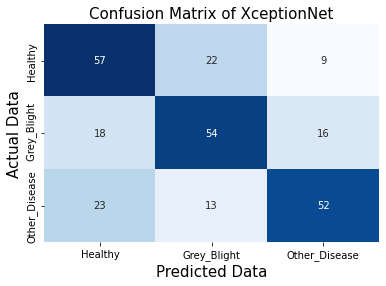


**Fig. 7:** Confusion matrix of XceptionNet

**Table 13:** Confusion matrix score of XceptionNet

|  | **TP** | **TN** | **FP** | **FN** |
| --- | --- | --- | --- | --- |
| **Healthy** | 57 | 135 | 41 | 31 |
| **Grey Blight** | 54 | 141 | 35 | 34 |
| **Other Disease** | 52 | 151 | 25 | 36 |

**Table 14:** Class-wise performance of XceptionNet

|  | **Accuracy** | **Precision** | **Recall** | **F-measure** | **Misclassification Rate** |
| --- | --- | --- | --- | --- | --- |
| **Healthy** | 74.29 | 58.16 | 64.77 | 61.29 | 25.71 |
| **Grey Blight** | 73.86 | 60.67 | 61.36 | 61.02 | 26.14 |
| **Other Disease** | 76.89 | 67.53 | 59.09 | 63.03 | 23.11 |
| **Average** | **75.01** | **62.12** | **61.74** | **61.78** | **24.99** |


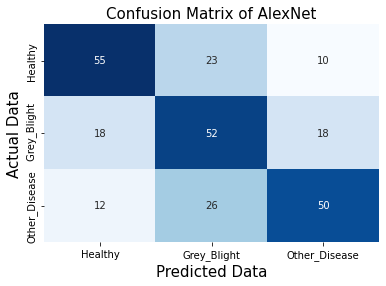


**Fig. 8:** Confusion matrix of AlexNet

**Table 15:** Confusion matrix score of AlexNet

|  | **TP** | **TN** | **FP** | **FN** |
| --- | --- | --- | --- | --- |
| **Healthy** | 55 | 146 | 30 | 33 |
| **Grey Blight** | 52 | 127 | 49 | 36 |
| **Other Disease** | 50 | 148 | 28 | 38 |

**Table 16:** Class-wise performance of AlexNet

|  | **Accuracy** | **Precision** | **Recall** | **F-measure** | **Misclassification Rate** |
| --- | --- | --- | --- | --- | --- |
| **Healthy** | 76.31579 | 64.71 | 62.5 | 63.58 | 23.68 |
| **Grey Blight** | 67.80303 | 51.49 | 59.1 | 55.03 | 32.2 |
| **Other Disease** | 75 | 64.1 | 56.82 | 60.24 | 25 |
| **Average** | **73.04** | **60.1** | **59.47** | **59.62** | **26.96** |


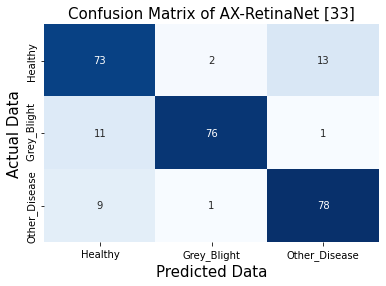


**Fig. 9:** Confusion matrix of AX-RetinaNet [33]

**Table 17:** Confusion matrix score of AX-RetinaNet [33]

|  | **TP** | **TN** | **FP** | **FN** |
| --- | --- | --- | --- | --- |
| **Healthy** | 73 | 156 | 20 | 15 |
| **Grey Blight** | 76 | 173 | 3 | 12 |
| **Other Disease** | 78 | 162 | 14 | 10 |

**Table 18:** Class-wise performance of AX-RetinaNet [33]

|  | **Accuracy** | **Precision** | **Recall** | **F-measure** | **Misclassification Rate** |
| --- | --- | --- | --- | --- | --- |
| **Healthy** | 86.74 | 78.49 | 82.95 | 80.66 | 13.26 |
| **Grey Blight** | 94.32 | 96.2 | 86.36 | 91.02 | 5.68 |
| **Other Disease** | 90.91 | 84.78 | 88.64 | 86.67 | 9.09 |
| **Average** | **90.66** | **86.5** | **85.98** | **86.12** | **9.34** |


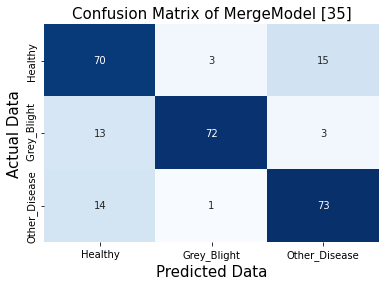


**Fig. 10:** Confusion matrix of MergeModel [35]

**Table 19:** Confusion matrix score of MergeModel [35]

|  | **TP** | **TN** | **FP** | **FN** |
| --- | --- | --- | --- | --- |
| **Healthy** | 70 | 149 | 27 | 18 |
| **Grey Blight** | 72 | 172 | 4 | 16 |
| **Other Disease** | 73 | 158 | 18 | 15 |

**Table 20:** Class-wise performance of MergeModel [35]

|  | **Accuracy** | **Precision** | **Recall** | **F-measure** | **Misclassification Rate** |
| --- | --- | --- | --- | --- | --- |
| **Healthy** | 82.95 | 72.16 | 79.55 | 75.68 | 17.05 |
| **Grey Blight** | 92.42 | 94.74 | 81.82 | 87.8 | 7.58 |
| **Other Disease** | 87.5 | 80.22 | 82.95 | 81.56 | 12.5 |
| **Average** | **87.63** | **82.37** | **81.44** | **81.68** | **12.37** |


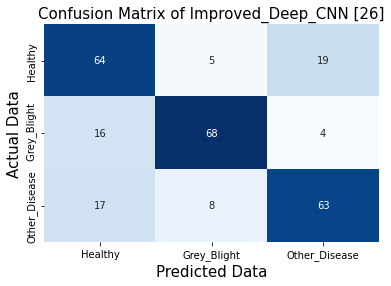


**Fig. 11:** Confusion matrix of Improved_Deep_CNN [26]

**Table 21:** Confusion matrix score of Improved_Deep_CNN [26]

|  | **TP** | **TN** | **FP** | **FN** |
| --- | --- | --- | --- | --- |
| **Healthy** | 64 | 143 | 33 | 24 |
| **Grey Blight** | 68 | 163 | 13 | 20 |
| **Other Disease** | 63 | 153 | 23 | 25 |

**Table 22:** Class-wise performance of Improved_Deep_CNN [26]

|  | **Accuracy** | **Precision** | **Recall** | **F-measure** | **Misclassification Rate** |
| --- | --- | --- | --- | --- | --- |
| **Healthy** | 78.41 | 65.98 | 72.73 | 69.19 | 21.59 |
| **Grey Blight** | 87.5 | 83.95 | 77.27 | 80.47 | 12.5 |
| **Other Disease** | 81.82 | 73.26 | 71.59 | 72.41 | 18.18 |
| **Average** | **82.58** | **74.40** | **73.86** | **74.03** | **17.42** |
